# Effects of graft detachment on the central corneal thickness after uncomplicated Descemet membrane endothelial keratoplasty

**DOI:** 10.1007/s00417-024-06452-7

**Published:** 2024-03-26

**Authors:** Tibor Lohmann, Sabine Baumgarten, David Kürten, Julia Prinz, Niklas Plange, Peter Walter, Matthias Fuest

**Affiliations:** Department of Ophthalmology, RWTH Aachen University, Pauwelsstrasse 30, 52074 Aachen, Germany

**Keywords:** Lamellar corneal surgery, Descemet membrane endothelial keratoplasty, Central corneal thickness, Graft detachment

## Abstract

**Purpose:**

To determine if early central corneal thickness (CCT) and best-corrected visual acuity (BCVA) changes indicate graft detachment after uncomplicated Descemet membrane endothelial keratoplasty (DMEK).

**Methods:**

In this analysis of our prospectively collected ADDA registry data (https://drks.de/search/de/trial/DRKS00027180), 45 pseudophakic eyes underwent DMEK surgery at the Department of Ophthalmology, RWTH Aachen University. Anterior segment optical coherence tomography (AS-OCT), the presence of stromal ripples on the posterior corneal surface, and BCVA measurements were assessed prior to, 1 day, 1 week, 1 month, and 6 months after surgery.

**Results:**

Eyes were categorized into three groups: no graft detachment (group 1) (20/45; 44.4%), < 1/3 graft detachment (group 2) (14/45; 31.1%), ≥ 1/3 graft detachment followed by rebubbling (group 3) (11/45; 24.4%). Eyes in group 3 had a greater CCT prior to (746.8 ± 95.8 µm vs. 665.0 ± 74.4 µm, *P* = 0.041), and 1 week (666.8 ± 119.5 µm vs. 556.5 ± 56.8 µm, *P* = 0.001) after DMEK compared to group 1. By 1 month, CCT in all groups aligned. Comparing prior to and 1 week after DMEK, none of the eyes in group 1 had an increase in CCT, while the CCT increased in 25.0% of eyes in group 2 and 22.2% in group 3. In group 1, 90.0% had a CCT of < 600 µm 1 week after DMEK, compared to only 50.0% in group 2 and 36.4% in group 3. In group 1, 90.0% (18/20) had an improved BCVA 1 week after DMEK, while in groups 2 and 3, 86.7% (12/14) and 18.2% (2/11) improved, respectively. One patient in group 3 showed posterior stromal ripples 1 day and 1 week after DMEK.

**Conclusion:**

If 1 week after uncomplicated DMEK CCT is < 600 µm and has decreased from before surgery, BCVA has improved, and there are no posterior stromal ripples, a graft detachment ≥ 1/3 and the need for rebubbling are very unlikely. In all other cases, meticulous slit-lamp and OCT inspection of the peripheral graft for detachments should be advised.


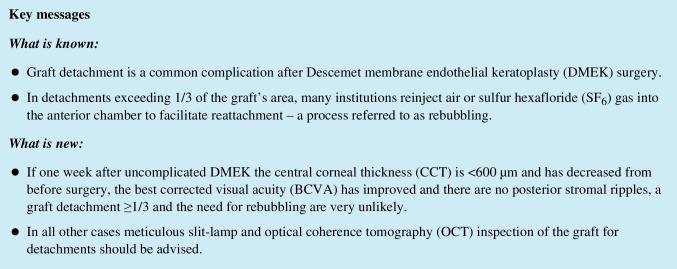


## Introduction

Descemet membrane endothelial keratoplasty (DMEK) has become the gold-standard treatment for corneal endothelial dysfunction such as Fuchs endothelial dystrophy (FED) or bullous keratopathy (BK), as it combines several major advantages over penetrating keratoplasty, including a quicker visual recovery, superior postoperative refractive outcomes, decreased rates of rejection, an increased postoperative wound strength, and better visual acuity results [1–4]. The technique was firstly described by Melles et al. in 2006 and subsequently modified to reduce complications and improve the surgical outcome [5–8].

One of the most common complications following DMEK surgery is graft detachment (2.0–82.0%) [1, 9–15]. The causes are multifactorial and include donor and recipient characteristics, such as donor and recipient age, as well as surgical parameters, such as graft folding and orientation and anterior chamber tamponade [16, 17]. Graft detachments predominantly occur in the first weeks after surgery, so patients must be monitored closely [18, 19]. When the area of graft detachment exceeds approx. 1/3, many institutions, us included, inject air or sulfur hexafloride (SF_6_) gas into the anterior chamber to facilitate reattachment [10, 15, 20, 21]. This procedure is referred to as *rebubbling*.

As peripheral detachments can be hard to detect and to make the ophthalmologist’s life easier, it would be very helpful to have week 1 parameters, which would strongly indicate full graft detachment, ideally by only looking at BCVA and a central anterior-segment optical coherence tomography (AS-OCT) scan.

Muijzer et al. established predictive biomarkers by applying a grid to AS-OCT images to divide the cornea in 25 corneal zones. While observing corneal thickness changes in all regions is time consuming, changes in the central corneal thickness (CCT) are quickly and reproducibly detected. Furthermore, another assumed predictive biomarker for graft detachment, the presence of posterior stromal ripples, irregularities in the posterior corneal profile that take the shape of a ripple, were evaluated [22]. Both CCT and presence of central stromal ripples can be detected in single central AS-OCT images and evaluated quickly in daily clinical practice.

In this study, we investigated the occurrence of graft detachment solely following uncomplicated DMEK surgery. The goal was to determine if early CCT, relative and absolute changes in CCT, the posterior corneal profile, and the best-corrected visual acuity (BCVA) translate into practical, clinical biomarkers for graft detachment.

## Materials and methods

### Study type

This retrospective analysis of the prospective ADDA registry was conducted by the Department of Ophthalmology, RWTH Aachen University GermanClinicalTrialsRegister. [23].

### Patient characteristics

The study included 45 eyes undergoing sole DMEK surgery (no combination with other procedures, e.g., cataract surgery (triple DMEK)). Mean age at the time of surgery was 75.1 ± 7.5 (60.9–86.7) years. Twenty-four (53.3%) were female, and 21 (46.7%) were male patients. Forty-three (95.4%) patients were Caucasian, and two (4.6%) were Asian. Surgery was performed on 26 (57.8%) right and 19 (42.2%) left eyes. Thirty-five (77.8%) eyes underwent DMEK because of FED, and ten (22.2%) for BK (Table 1). All patients were pseudophakic. All patients had uncomplicated cataract surgery with in the bag posterior intraocular lens implantation at least 3 months prior to DMEK surgery.

**Table 1:** Characteristics of patients receiving uncomplicated Descemet membrane endothelial keratoplasty (DMEK)

Characteristics:	
Sex:	
Male	21 (46.67%)
Female	24 (53.33%)
Race:	
Caucasian	43 (95.35%)
Asian	2 (4.65%)
Right eye	26 (57.78%)
Left eye	19 (42.22%)
CCT (µm) preDMEK	686.28 ± 87.79 (519.00–945.00)
BCVA (logMAR) preDMEK	0.84 ± 0.43 (0.30–2.00)

**Fig. 1:**
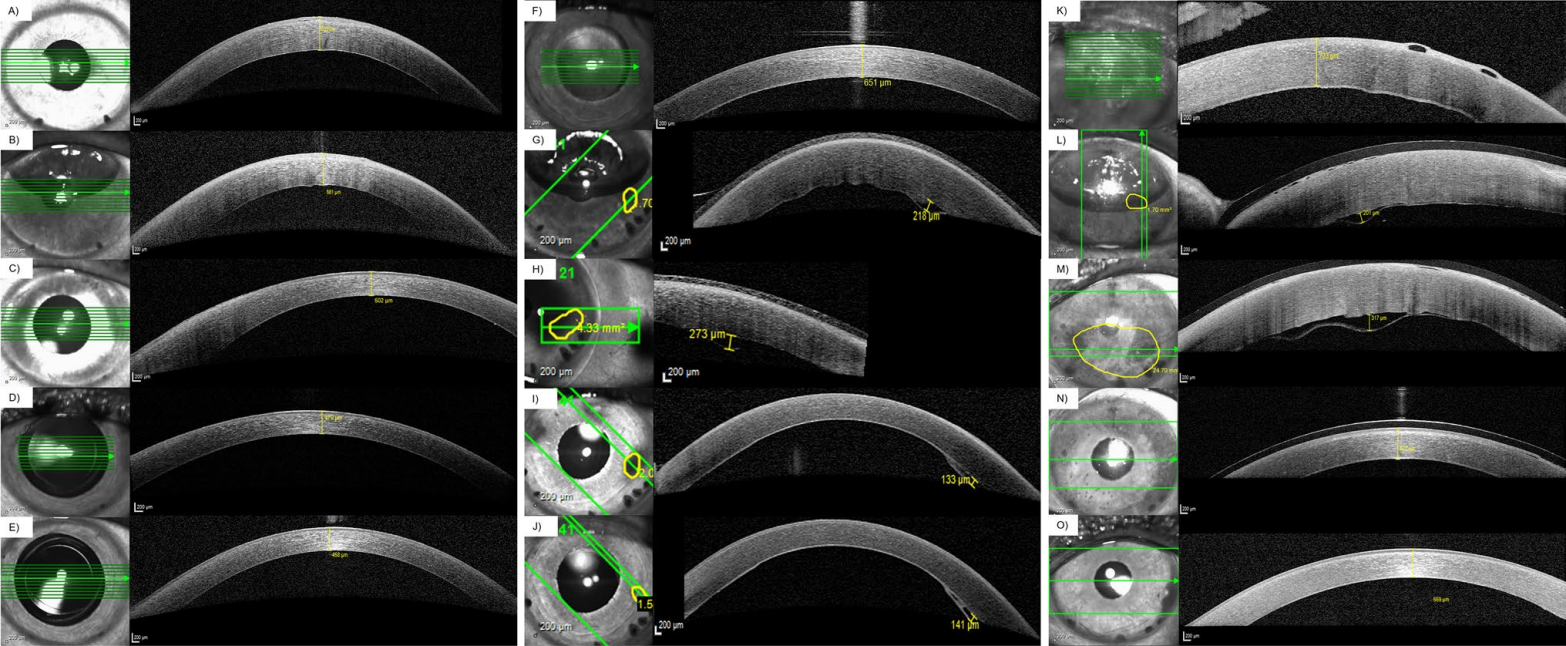
Central corneal thickness (CCT), area of detachment, and maximum distance of detachment measurements prior to, 1 day, 1 week, 1 month, and 6 months after Descemet membrane endothelial keratoplasty (DMEK) via anterior segment spectral-domain optical coherence tomography (AS-OCT, Heidelberg Engineering, Heidelberg, Germany). **A**–**E** No detachment. **F**–**J** Graft detachment < 1/3 of graft area. **K**–**O** Graft detachment ≥ 1/3 of graft area with subsequent rebubbling. M: Encircled area of detachment in the en face image on the left. Proportional detachment ≥ 1/3 of the graft’s area

**Fig. 2:**
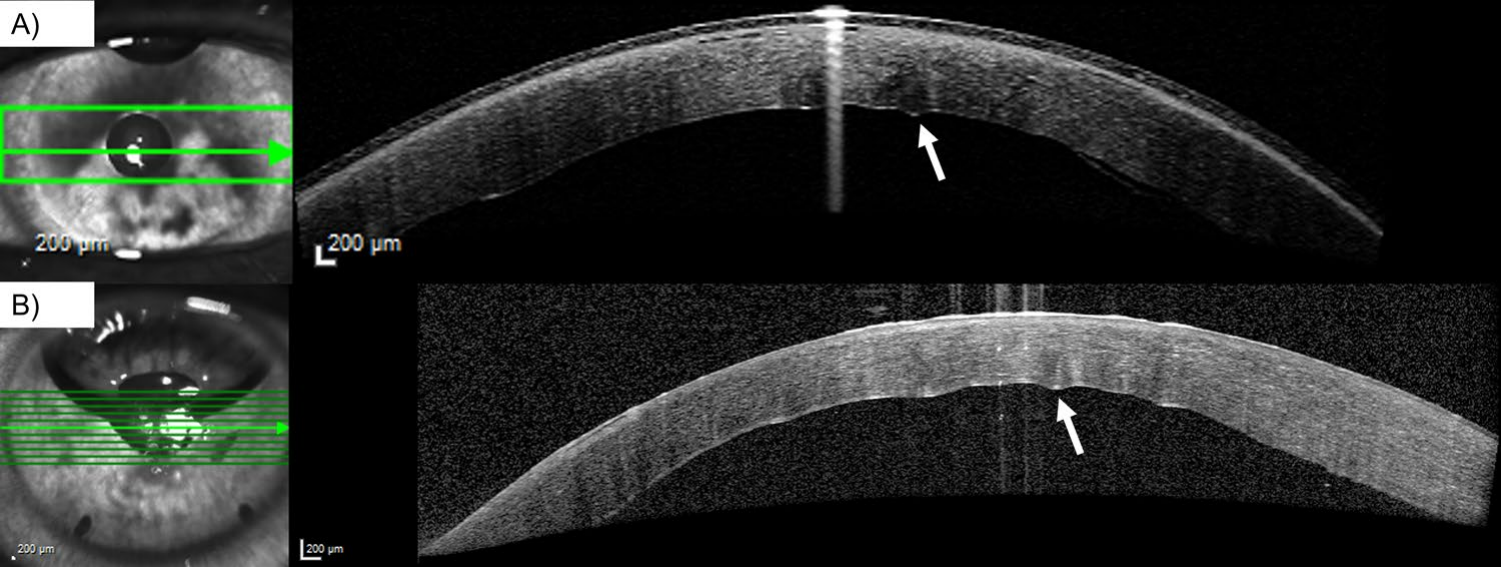
Presence of posterior stromal ripples after Descemet membrane endothelial keratoplasty (DMEK) via anterior segment spectral-domain optical coherence tomography (AS-OCT, Heidelberg Engineering, Heidelberg, Germany). **A** Mild posterior stromal ripples 1 week after DMEK. White arrow indicates posterior stromal ripple. **B** Severe posterior stromal ripples 1 day after DMEK. White arrow indicates posterior stromal ripple

Graft detachment was defined as a gap between the donor graft and the recipient corneal stroma observed in slit lamp examinations and confirmed via AS-OCT (Fig. 1; Spectralis-OCT, Heidelberg Engineering GmbH, Heidelberg, Germany). The area of detachment was encircled in the en face images using the Spectralis-OCT software, as seen in Fig. 1M. By dividing the encircled area of detachment by the total graft area, the proportional detachment was obtained. Graft detachments were subdivided into minor (< 1/3 of the graft) or major (≥ 1/3 of the graft) detachments, with a subsequent indication for a rebubbling (Fig. 1). A successful rebubbling was defined by a complete graft reattachment at the next follow-up. In a modified classification based on the work of Coco et al., the degree of posterior stromal ripples was graded as none, mild, or severe based on their number (0 for none, ≤ 4 for mild, or > 4 for severe) as presented in the single most central cross-sectional AS-OCT image (parameters in Spectralis-OCT: 20° × 10°, 512 A-scans, 41 sections at 139-µm width), as seen in Fig. 2 [22]. To match the term used in the original publication by Coco et al. firstly addressing the appearance of corneal ripples and their relevance for DMEK graft detachments, we choose to use the term *posterior stromal ripples*, instead of the also commonly used term *stromal folds.*

### Inclusion criteria

Included were pseudophakic patients undergoing uncomplicated sole DMEK surgery without any intraoperative (e.g., iris damage, bleeding, or additional intraoperative iridectomies) or postoperative complications (e.g., intraocular pressure (IOP) spikes, pronounced intraocular inflammation, and/or fibrin deposition). Rebubbling was not considered a complication.

### Exclusion criteria

Excluded were eyes with additional eye diseases apart from FED or BK. Eyes with any prior ocular surgery apart from cataract surgery were also excluded. The eyes received no preoperative eye drops apart from lubricating and/or antibiotic eye drops in case of progressed BK.

### Surgical technique

Surgery was performed as previously described [15]. Briefly, two yttrium–aluminum-garnet laser (Visulas YAG II, Carl Zeiss Meditec AG, Jena, Germany) iridotomies were performed inferiorly (usually at 5 and 7 o’clock) at least 24 h prior to DMEK surgery. No intraoperative iridectomies were performed. Transplants were prestripped less than 24 h prior to surgery. DMEK surgery was performed as previously described by Melles et al. [5, 6]. All DMEK surgeries were performed under general anesthesia. Donor grafts had a median diameter of 8.0 (7.25–9.0) mm. The central host DM was stripped under air aiming for a diameter approximately 1.0 mm larger than the donor graft. After buffered saline solution exchange (BSS, Alcon, Fort Worth, USA), the stained (trypan blue, VisionBlue, DORC, Rotterdam, Netherlands) donor graft was injected into the anterior chamber. By carefully impressing and tapping the corneal surface with a shallow anterior chamber, all grafts could be unfolded. An air bubble was injected behind the graft to fixate it. The anterior chamber was then fully filled with SF_6_ 20.0% (Arceole pure SF_6_, Arcadophta, Toulouse, France). Intraocular pressure (IOP) was estimated by indentation and set to normal levels. In case of low IOP, additional SF_6_ 20.0% was injected, in high IOP released. Finally, a contact lens was placed and dexamethasone dihydrogen phosphate disodium 1.0 mg/ml and gentamicin sulfate 5.0 mg/ml eye drops (Dexa-Gentamicin, Ursapharm, Saarbrücken, Germany) and pilocarpine 20.0 mg/g eye drops (Pilomann 2%, Bausch Lomb, Rochester, USA) were applied.

Rebubbling was performed in supine position under local anesthesia, when more than one-third of the graft was detached between 1 and 4 weeks after DMEK surgery. Following a 23G paracentesis, SF_6_ 20.0% gas was injected to fill the anterior chamber. IOP was checked and a contact lens placed followed by dexamethasone dihydrogen phosphate disodium 1.0 mg/ml and gentamicin sulfate 5.0 mg/ml eye drops and pilocarpine 20.0 mg/g eye drops.

### Medication

For the first week after DMEK surgery, patients received dexamethasone dihydrogen phosphate disodium 1.0 mg/ml and gentamicin sulfate 5.0 mg/ml eye drops five times daily and prednisolone acetate 10.0 mg/ml eye drops (Inflanefran forte, Allergan, Dublin, Ireland), five times daily. Additionally, patients received pilocarpine 20.0 mg/g eye drops twice daily until complete resolution of intraocular 20.0% SF_6_ gas, respectively. After the first month, only prednisolone acetate 10.0 mg/ml eye drops were continued. These were tapered by one drop every month to a maintenance dose of once daily for life.

### Examination and follow-up

Eye examinations prior to surgery, 1 day, 1 week, 1 month, and 6 months after surgery were analyzed. This study evaluated CCT measurements via AS-OCT including CCT values, absolute and relative changes in CCT, and area and maximum distance of detachment as well as irregularities in the posterior corneal profile in the shape of ripples. The BCVA was measured using the Snellen visual acuity chart, and we analyzed the results using logarithm of the minimum angle of resolution (logMAR) equivalent units.

### Ethics

This study followed the tenets of the Declaration of Helsinki and was approved by the Institutional Ethical Review Board of the RWTH Aachen University. This study was conducted as part of the ADDA registry [23].

### Statistics

If not specified, otherwise all values were expressed as the mean ± standard deviation (range min–max). All statistical analyses were performed with GraphPad Prism (GraphPad Prism V9, San Diego, USA). Comparisons between categorical variables were conducted using the Fisher’s exact test or *X*^2^-test for multiple comparisons. For continuous measures, the paired and unpaired *t*-tests or simple one-way ANOVA were used. For values not following a normal Gaussian distribution, Mann–Whitney-*U* or Wilcoxon signed-rank test was used. A *P* value of < 0.05 was considered statistically significant.

## Results

No significant difference in age, sex, race, or indication for DMEK surgery comparing the three groups mentioned above was observed (Table 2). Twenty-five (55.6%) of 45 eyes showed a graft detachment. In eleven eyes (24.4%), detachment was ≥ 1/3 of the graft’s area subsequently leading to rebubbling. Mean time to rebubbling was 10.5 ± 4.5 (7.0–22.0) days. No eyes received more than one rebubbling. To further evaluate the CCT in association with graft detachment, patients were divided into three groups: no graft detachment (group 1), graft detachment < 1/3 of the area (group 2), and graft detachment ≥ 1/3 of the area, subsequently needing a rebubbling (group 3). One day after surgery, six of 14 patients (42.9%) in group 2 and five of eleven patients (45.5%) in group 3 showed detachment. One week after surgery, 14 of 14 patients (100.0%) in group 2 and eleven of eleven patients (100.0%) in group 3 showed detachment. One month after surgery, eleven of 14 patients (78.6%) in group 2 and two of eleven patients in group 3 showed detachment. Six months after surgery, one of 14 patients in group 2 (18.2%) and no patient in group 3 showed detachment (Table 2).

**Table 2:** Characteristics of patients receiving uncomplicated Descemet membrane endothelial keratoplasty (DMEK) in three groups

Characteristics:	Group 1: no detachment (*N* = 20/45 (79.4%))	Group 2: detachment < 1/3 (*N* = 14/45 (21.6%))	Group 3: detachment ≥ 1/3 + rebubbling (*N* = 11/45 (24.4%))	*P*-value
Age (years)	74.41 ± 7.17 (62.03–87.25)	74.80 ± 8.55 (60.85–86.74)	76.34 ± 6.14 (68.08–85.94)	0.836
Sex:				
Male	10 (50.00%)	7 (50.00%)	4 (36.36%)	0.733
Female	10 (50.00%)	7 (50.00%)	7 (63.64%)	
Indication:				
FED	15 (75.00%)	12 (85.71%)	8 (72.7%)	0.683
BK	5 (25.00%)	2 (14.29%)	3 (27.3%)	
Race:				
Caucasian	20 (100.00%)	14 (100.00%)	9 (81.81%)	> 0.999
Asian	0	0	2 (18.19%)	
Right eye	13 (65.00%)	7 (50.00%)	6 (54.55%)	0.663
Left eye	7 (35.00%)	7 (50.00%)	5 (45.45%)	
Time to rebubbling (days)	N/A	N/A	10.45 ± 4.54 (7.00–22.00)	
BCVA (logMAR)				
PreDMEK	0.83 ± 0.45 (0.30–2.00)*	0.82 ± 0.34 (0.40–2.00)	0.88 ± 0.53 (0.52–2.00)*	* < 0.001
1 week	0.45 ± 0.21 (0.22–1.00)	0.66 ± 0.54 (0.10–1.30)	1.03 ± 0.21 (0.80–1.30)*	
1 month	0.36 ± 0.23 (0.10–1.00)	0.41 ± 0.25 (0.00–0.80)	0.56 ± 0.30 (0.01–1.00)	
3 months	0.26 ± 0.16 (0.10–0.60)	0.24 ± 0.10 (0.10–0.30)	0.24 ± 0.16 (0.00–0.40)	
6 months	0.18 ± 0.07 (0.10–0.21)	0.40 ± 0.18 (0.00–0.40)	0.21 ± 0.14 (0.00–0.40)	
CCT (µm)				
PreDMEK	665.00 ± 74.42 (519.00–881.00)*	672.50 ± 85.44 (569.00–868.00)	746.80 ± 95.84 (627.00–945.00)*	*0.041
1 day	660.80 ± 78.28 (494.00–767.00)	650.80 ± 102.50 (508.00–812.00)	702.00 ± 88.85 (599.00–856.00)	
1 week	556.50 ± 56.84 (443.00–687.00)*	603.40 ± 81.83 (473.00–772.00)	666.80 ± 119.50 (476.00–882.00)*	* < 0.001
1 month	519.60 ± 28.76 (468.00–586.00)	538.60 ± 40.58 (481.00–608.00)	578.40 ± 82.21 (440.00–733.00)	
6 months	502.30 ± 22.67 (456.00–545.00)	513.60 ± 24.08 (478.00–550.00)	538.80 ± 49.87 (450.00–617.00)	
Area of detachment (mm^2^)				
1 day	N/A	1.84 ± 1.94 (0.00–5.46)*	7.15 ± 7.86 (0.00–18.57)*	* < 0.001
1 week		5.54 ± 2.39 (2.50–9.56)*	23.39 ± 6.09 (11.15–30.40)*	*0.027
1 month		3.10 ± 3.18 (0.00–10.40)	1.060 ± 2.26 (0.00–6.14)	
6 months		0.11 ± 0.41 (0.00–1.54)	0.00 ± 0.00 (0.00–00.00)	
Maximum distance of detachment (µm)				
1 day	N/A	134.89 ± 123.43 (0.00–322.00)	174.60 ± 159.58 (0.00–302.00)	
1 week		230.23 ± 90.36 (126.00–391.00)*	512.30 ± 239.59 (290.00–1110.00)*	* < 0.001
1 month		167.92 ± 127.36 (0.00–349.00)*	62.00 ± 132.82 (0.00–360.00)*	*0.037
6 months		10.07 ± 37.68 (0.00–141.00)	0.00 ± 0.00 (0.00–00.00)	
CCT change (relative in % of initial CCT)				
PreDMEK–1 week	84.63 ± 10.42 (58.57–98.42)	91.92 ± 118.37 (65.08–133.3)	90.56 ± 15.70 (64.32–122.0)	
1 week–1 month	94.02 ± 7.58 (76.71–110.20)*	90.39 ± 10.86 (74.35–108.92)	79.59 ± 10.39 (69.05–94.02)*	*0.006
CCT change (absolute in µm decrease)				
PreDMEK–1 week	− 108.50 ± 86.29 (− 11.00 to − 365.00)	64.23 ± 125.61 (193.00 to − 271.00)	70.80 ± 115.01 (159.00 to − 264.00)	
1 week–1 month	36.84 ± 45.15 (45.00 to − 160.00)	64.79 ± 72.78 (45.00 to − 198.00)	93.60 ± 77.39 (− 25.00–255.00)	
Presence of posterior stromal ripples				
PreDMEK				
Mild	3	3	2	0.566
Severe	3	1	3	
1 day				
Mild	6	3	3	> 0.999
Severe	1	0	1	
1 week				
Mild	2	2	2	> 0.999
Severe	0	1	1	

* represents a *P*-value < 0.05

**Fig. 3:**
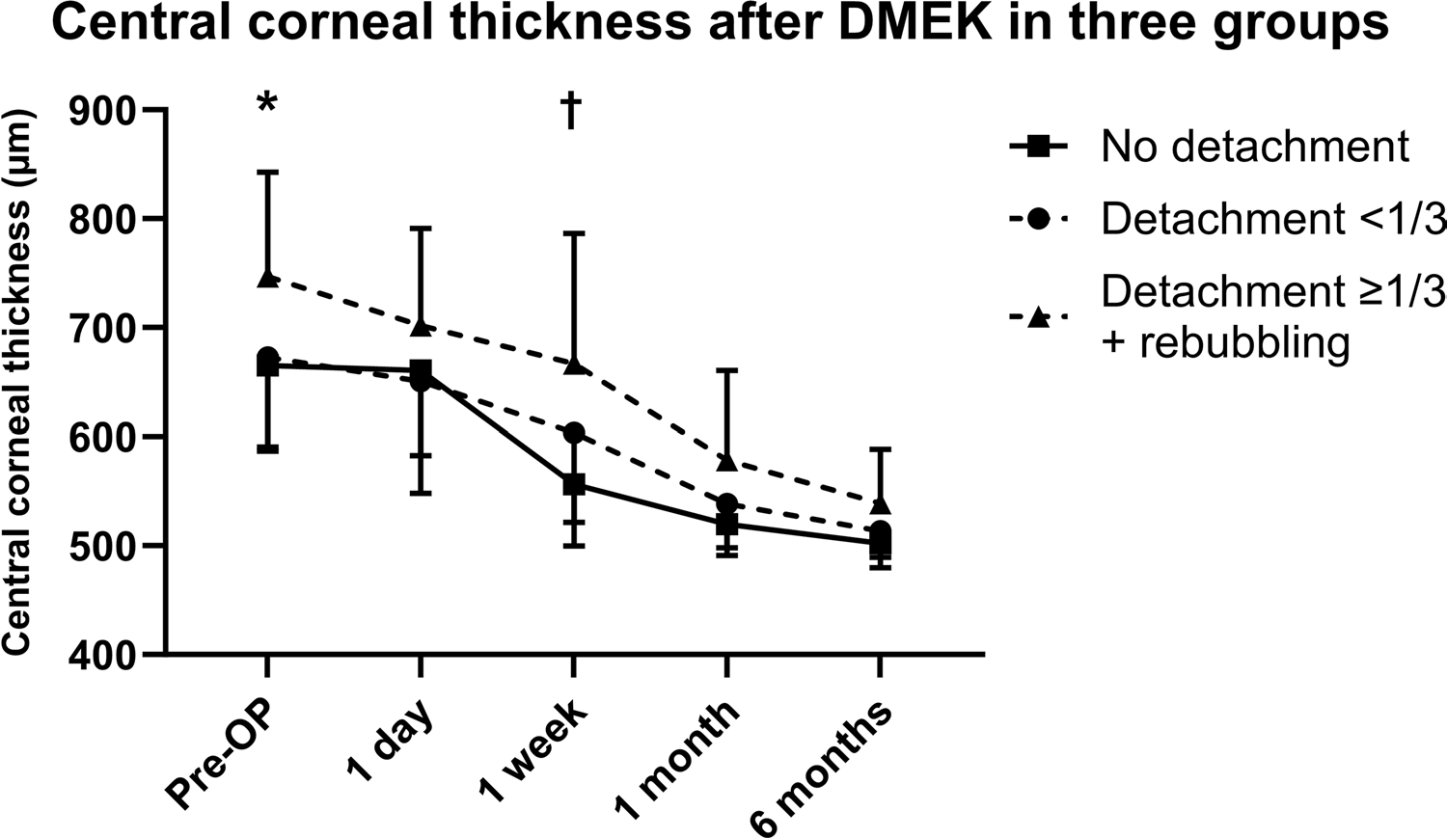
Central corneal thickness (CCT) prior to and after Descemet membrane endothelial keratoplasty (DMEK) at various time points in µm in three groups (no detachment: no graft detachment; detachment < 1/3: graft detachment < 1/3 of graft area; detachment ≥ 1/3 + rebubbling: graft detachment ≥ 1/3 of graft area with subsequent rebubbling). Error bars indicate standard deviation (SD). *No detachment vs. detachment ≥ 1/3 + rebubbling: *P* = 0.041; †no detachment vs. detachment ≥ 1/3 + rebubbling: *P* = 0.001

Eyes in group 3 had a greater CCT prior to (746.8 ± 95.8 (627.0–945.0) µm vs. 665.0 ± 74.4 (519.0–881.0) µm, *P* = 0.041), and 1 week (666.8 ± 119.5 (476.0–882.0) µm vs. 556.5 ± 56.8 (443.0–687.0) µm, *P* = 0.001) after DMEK surgery compared to eyes in group 1 (Fig. 3, Table 2). After 1 month, CCT in all groups aligned (Fig. 3).

In group 1, 90.0% (2/20) of eyes had a CCT of less than 600.0 µm 1 week after DMEK, while only 50.0% (7/14) in group 2 and 36.4% (4/11) in group 3 were less than 600.0 µm. In group 1, maximum CCT 1 week after DMEK was 678.0 µm, while maximum CCT in groups 2 and 3 was 772.0 µm and 882.0 µm, respectively.

**Fig. 4:**
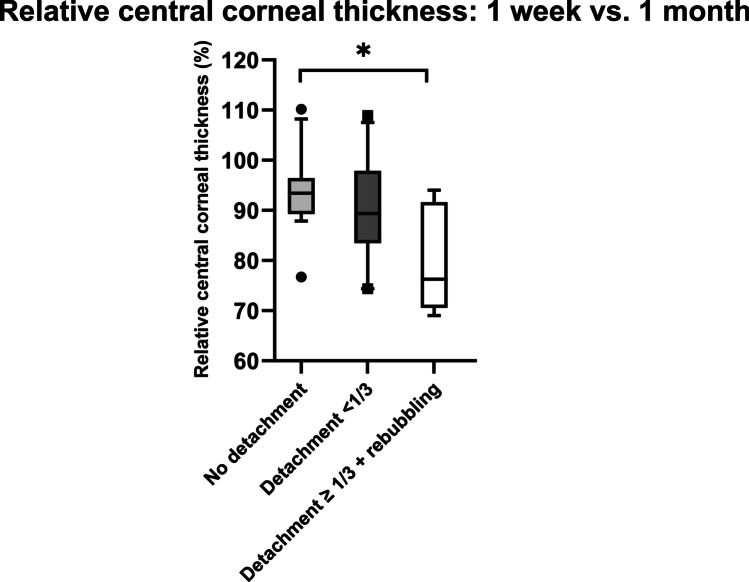
Relative central corneal thickness (CCT) 1 week vs. 1 month after Descemet membrane endothelial keratoplasty (DMEK) in three groups (no detachment: no graft detachment; detachment < 1/3: graft detachment < 1/3 of graft area; detachment ≥ 1/3 + rebubbling: graft detachment ≥ 1/3 of graft area with subsequent rebubbling). Median is indicated with horizontal line the box. Bottom of the box represents first quartile and top third quartile. Whiskers indicate 10th to 90th percentile. Outliers are indicated with black dots. *P = 0.006

Comparing prior to and 1 week after DMEK, none of the eyes in group 1 showed an increase in CCT, while 25.0% of eyes in group 2 and 22.2% in group 3 showed an increase in CCT over this time period (Table 2). The relative change in CCT was greater from 1 week to 1 month comparing group 1 and group 3 (94.0 ± 7.6 (76.7–110.2) % vs. 79.6 ± 10.4 (69.1–94.0) %; Fig. 4).

**Fig. 5:**
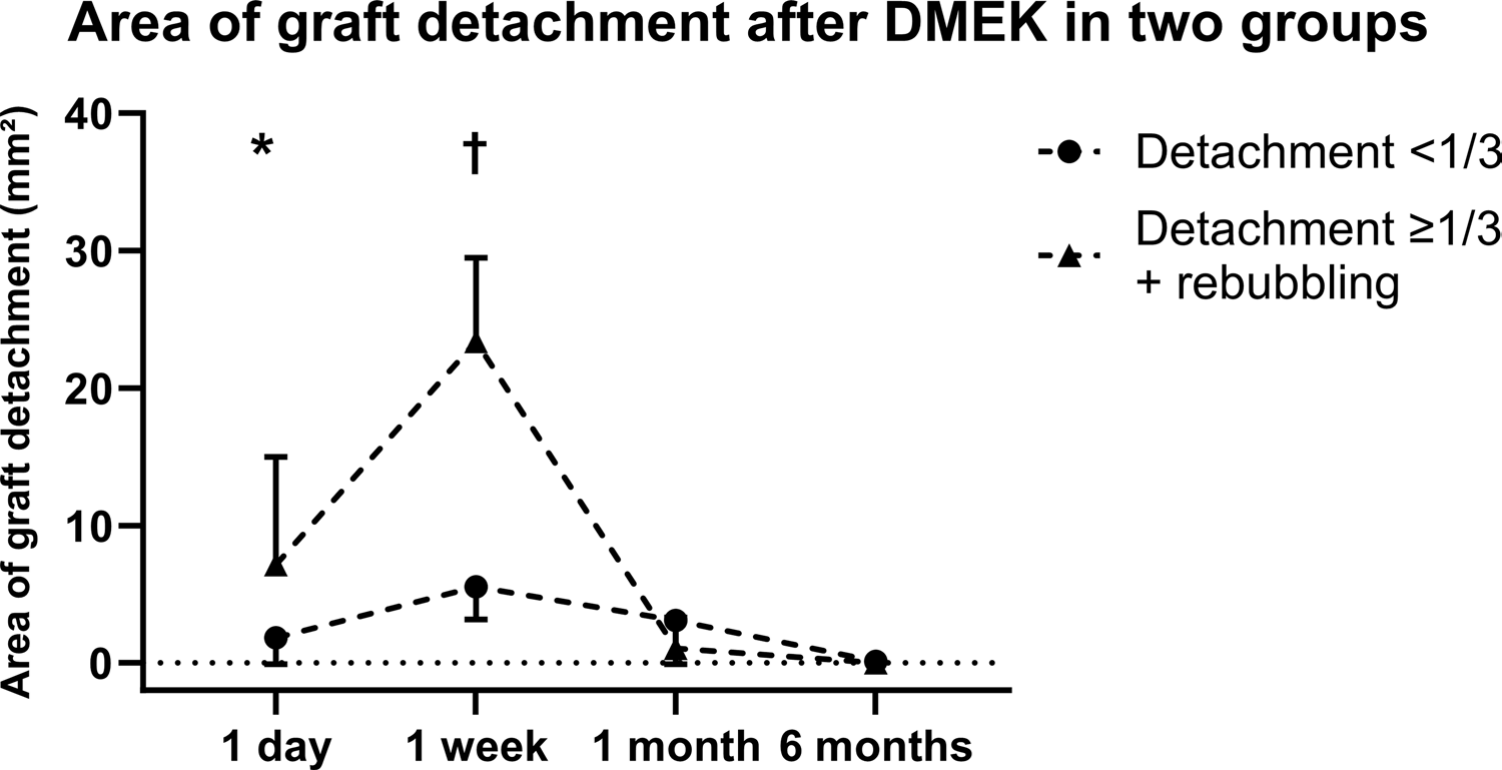
Area of graft detachment after Descemet membrane endothelial keratoplasty (DMEK) at various time points in µm in two groups (detachment < 1/3: graft detachment < 1/3 of graft area; detachment ≥ 1/3 + rebubbling: graft detachment ≥ 1/3 of graft area with subsequent rebubbling). Error bars indicate standard deviation (SD). *Detachment < 1/3 vs. detachment ≥ 1/3 + rebubbling: *P* < 0.001; †detachment < 1/3 vs. detachment ≥ 1/3 + rebubbling: *P* = 0.027

**Fig. 6:**
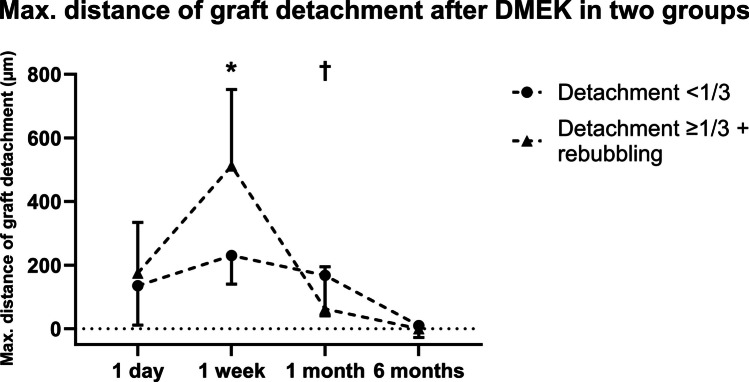
Maximum distance of graft detachment after Descemet membrane endothelial keratoplasty (DMEK) at various time points in µm in two groups (detachment < 1/3: graft detachment < 1/3 of graft area; detachment ≥ 1/3 + rebubbling: graft detachment ≥ 1/3 of graft area with subsequent rebubbling). Error bars indicate standard deviation (SD). *Detachment < 1/3 vs. detachment ≥ 1/3 + rebubbling: *P* < 0.001; †detachment < 1/3 vs. detachment ≥ 1/3 + rebubbling: *P* = 0.037

Comparing the area of graft detachment in groups 2 and 3, a greater detached area was measured one day (1.8 ± 1.9 (0.0–5.5) mm^2^ vs. 7.2 ± 7.9 (0.0–18.6) mm^2^, *P* < 0.001) and 1 week after surgery (5.5 ± 2.4 (2.5–9.6) mm^2^ vs. 23.4 ± 6.1 (11.2–30.4) mm^2^, *P* = 0.027) in group 3 (Fig. 5, Table 2). Examining the maximum distance of the transplant to the host stroma in graft detachments in groups 2 and 3, the distance was greater in group 3 after 1 week (230.2 ± 90.4 (126.0–391.0) µm vs. 512.3 ± 239.6 (290.0–1110.0) µm, *P* < 0.001), while after 1 month, it was greater in group 2 (167.9 ± 127.4 (0.0–349.0) µm vs. 62.0 ± 132.8 (0.0–360.0) µm, *P* = 0.037; Fig. 6, Table 2).

Posterior stromal ripples were present in all groups prior to DMEK surgery (Table 2). A difference in the presence of posterior stromal ripples from before to 1 day and 1 week after DMEK surgery was not found (*P* > 0.999). Severe posterior stromal ripples both 1 day and 1 week after surgery were only seen in a patient in group 3.

**Fig. 7:**
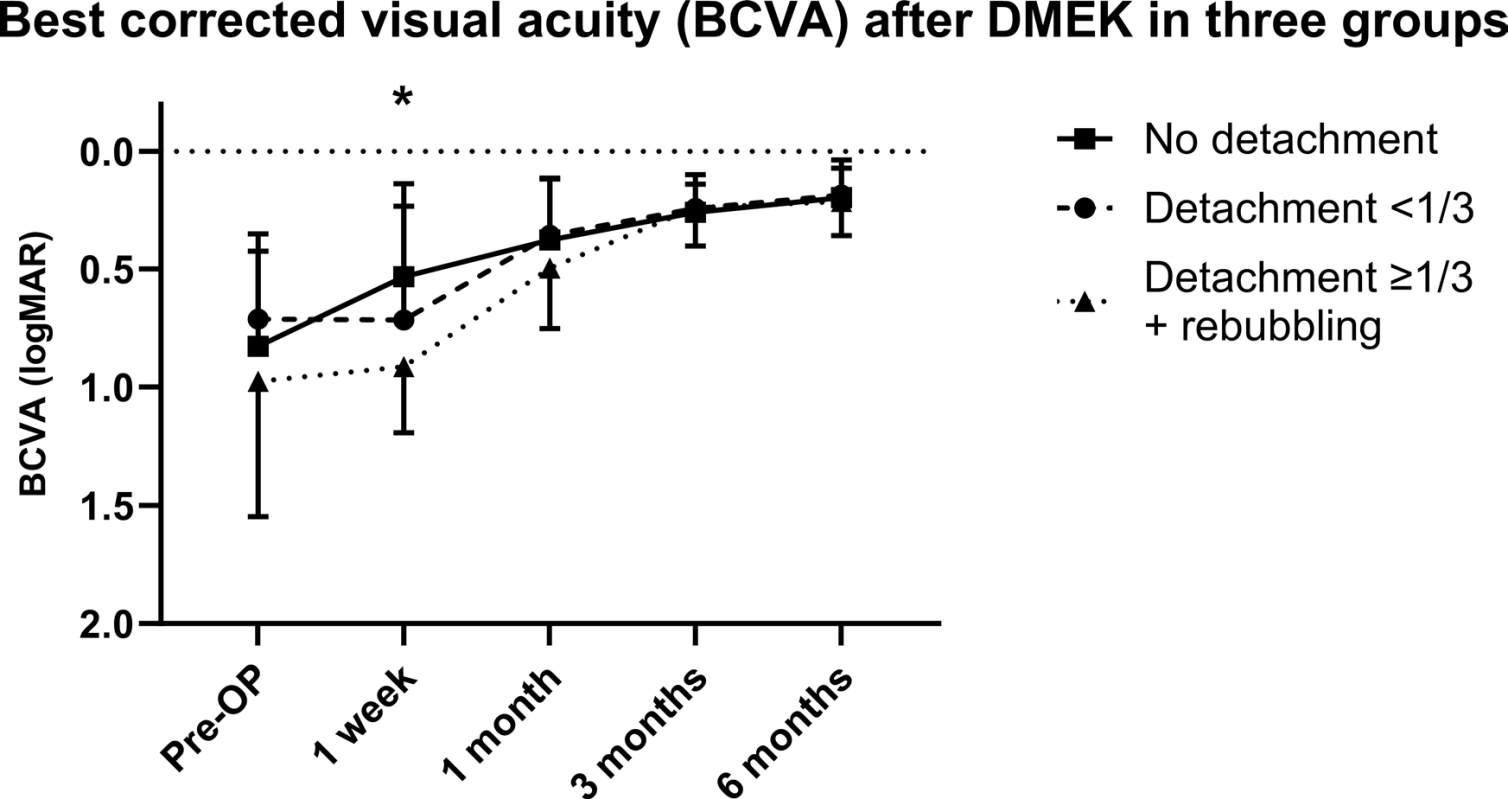
Best-corrected visual acuity after Descemet membrane endothelial keratoplasty (DMEK) at various time points in µm in two groups (detachment < 1/3: graft detachment < 1/3 of graft area; detachment ≥ 1/3 + rebubbling: graft detachment ≥ 1/3 of graft area with subsequent rebubbling). Error bars indicate standard deviation (SD). *No detachment vs. detachment ≥ 1/3 + rebubbling: *P* < 0.001

The BCVA was better in group 1 compared to group 3 1 week after DMEK surgery (0.45 ± 0.21 (0.22–1.00) logMAR vs. 1.03 ± 0.21 (0.80–1.30) logMAR, *P* = 0.002; Fig. 7, Table 2). In group 1, 90.0% (18/20) showed better BCVA 1 week after DMEK, while in groups 2 and 3, 86.7% (12/14) and 18.2% (2/11) improved in BCVA. In all groups, BCVA was 0.19 ± 0.11 (0.00–0.40) logMAR at the last visit 6 months after surgery.

## Discussion

Graft detachment is a common complication after DMEK surgery [24, 25]. The detection of an early graft detachment after DMEK surgery is essential for a successful outcome as it might demand a rebubbling [1, 19]. In this study on 45 eyes undergoing uncomplicated DMEK surgery, 25 (55.6%) showed graft detachment, with 11 (24.4%) eyes needing rebubbling due to detachment ≥ 1/3 of the graft’s area (group 3). Those eyes had a greater CCT prior to and 1 week after DMEK compared to eyes without any detachment (group 1). One week after uncomplicated DMEK, 90.0% of patients in group 1 had a CCT smaller than 600.0 µm and an improved BCVA. A CCT greater than 700.0 µm and an increase in CCT at 1 week only occurred in graft detachment (groups 2 and 3). Stromal ripples were present in all groups prior to DMEK surgery; severe posterior stromal ripples 1 day and 1 week after surgery were only present in a patient in group 3.

While the reported occurrence of graft detachments varies in the literature (2.0–82.0%) [1, 9–14], our findings match those reported by Muijzer et al. (50.8% graft detachment, 26.2% rebubbling) and Guindolet et al. (36.0% rebubbling) [26, 27]. Yeh et al. also reported overall detachment of approx. 50.0% over the course of 6 months, with 33.0% showing graft detachment of more than 1/3 of the graft’s area [28]. Differences in reported detachment rates could relate to possible risk factors reported in the literature, including donor characteristics, such as donor age or low endothelial cell density; recipient factors, such as recipient age; and surgical parameters, such as descemetorhexis diameter, graft decentration, anterior chamber tamponade agent, postoperative intraocular pressure, and surgeon experience [16]. Overall, agreement on these risk factors across reports was weak [16].

Reports on the CCT 1 week after DMEK surgery are scarce. Guindolet et al. reported that the preoperative CCT was not significantly different between the group that required rebubbling (624 ± 92 µm) compared to the one that did not (660.0 ± 120.0 µm; *P* = 0.340) [27]. Contrarily, we found greater preoperative CCT values in eyes in group 3 (746.8 ± 95.8 µm, group 3) compared with eyes in group 1 (665.0 ± 74.4 µm, group 1; *P* = 0.041). While CCT in eyes without rebubbling matched those in our study without any detachment, CCT in eyes with rebubbling differed greatly. This could have been caused by a wider variety of included patients, since only graft preparation failures and inverted grafts were excluded [27]. CCT measurements 1 week after DMEK were not reported [27].

Coco et al. reported absolute CCT values at approx. 3 days after DMEK only in relation to the presence of stromal ripples, not in relation to graft detachment nor subsequent rebubbling (no stromal ripples: 624.3 ± 80.2 μm; mild stromal ripples: 707.6 ± 62.9 μm; moderate/severe stromal ripples: 757.4 ± 125.1 μm; *P* < 0.001 one-way ANOVA) [22]. While an exact comparison to our data is impossible, the CCT range matched our findings.

Muijzer et al. performed pachymetry mapping showing an increase in local corneal thickness in zones with a graft detachment [26]. One day after surgery, the mean corneal thickness over all zones was higher in eyes that developed a graft detachment compared with subjects without a graft detachment (745.0 ± 82.4 µm vs. 805.4 ± 98.03 µm, *P* = 0.015) [26]. Importantly, within subjects who developed a graft detachment, the corneal zones in which the graft detached were thicker compared with zones in which the graft remained attached; conversely, the thickness of corneal zones in which the graft remained attached in the detachment group did not differ compared with the corneal thickness in subjects without a graft detachment [26]. While predictability of graft detachment using this method was valid, no correlation to the necessity for rebubbling was made [26]. Furthermore, absolute values on the CCT were not reported for any time [26]. Dirisamer et al. found that corneal thickness was increased in corneal quadrants with detached grafts compared with adjacent corneal quadrants with attached grafts [29]. A greater CCT in eyes with graft detachment as detected in our data supports this finding.

In our study, a relative and absolute increase in CCT after 1 week was only present in eyes in groups 2 and 3. Interestingly, Guindolet et al. reported that a decrease in the CCT after 1 day was associated with a lower risk for rebubbling, and that an increase of 20.0% of the CCT was associated with an increased risk for rebubbling [27]. This is supported by our data on the 1-week examination, where a decrease in relative/absolute CCT and a CCT of less than 700 µm was associated with graft attachment without the need for rebubbling. In a subgroup analysis regarding preoperative CCT, Guindolet et al. showed no difference in the rebubbling rate between patients with CCT greater than 700.0 µm and those with a CCT less than 700.0 µm [27]. While in our study the CCT was greater in eyes in group 3 compared to eyes in group 1, all groups contained eyes with CCT greater 700.0 µm prior to surgery. Guindolet et al. suggested introducing indices of CCT increase to predict a detachment risk in clinical practice [27].

Our study showed that in groups 2 and 3, the area and maximum detachment distance increased from 1 day to 1 week after surgery, followed by a consecutive decrease to 6 months after surgery. In a prospective study, Yeh et al. described a “biphasic” adherence pattern: initial attachment within the first hour after surgery in the majority of eyes, followed by a partial detachment in approx. 1/3 of the eyes at 1 week and a recovery of graft adherence at 1 to 6 months [28]. In eyes with less than 1/3 of the graft’s area detached, we found similar results: both max. area and distance of detachment were largest 1 week after surgery and decrease afterwards. Also, our data agreed with a 100.0% negative predictive value of AS-OCT scans 1 week and 1 month after DMEK surgery: If graft attachment (group 1) or graft detachment in less than 1/3 of the graft’s area (group 2) was recorded, a detachment of over 1/3 of the area did not occur after 6 months [28]. Hence, the 1-week and 1-month postoperative AS-OCT scan showed good sensitivity. Spontaneous graft detachment after 1 month after DMEK was not reported by groups previously mentioned, yet rare cases of late graft detachment exist [30]. We would argue that in cases of good visual recovery and inconspicuous slit-lamp examination, a AS-OCT is not mandatory on examinations past 1 month after surgery.

In a study on 111 eyes receiving DMEK surgery, Kramer et al. found a difference of BCVA improvement of 0.22 logMAR in favor of eyes with spontaneous graft adherence without intervention compared to eyes needing rebubbling 6 months after surgery (*P* = 0.048) [31]. While in our study no difference was found after 6 months, BCVA was higher 1 week after DMEK surgery in eyes without detachment (group 1) compared to eyes receiving rebubbling (group 3, *P* < 0.001). One month after surgery, no difference between groups was found, hinting at a quick recovery in eyes receiving rebubbling. Yeh et al. reported 44.0% of eyes receiving rebubbling reached a BCVA better or equal to approx. 0.2 logMAR 6 months after DMEK, while in our study, 75.0% of patients receiving rebubbling reached a BCVA better or equal to approx. 0.2 logMAR 6 months after surgery. Overall, a swift and excellent visual recovery has been described for DMEK, with and without rebubbling [15, 32, 33]. Furthermore, Dunker et al. found no relationship between the timing of rebubbling (i.e., within 1 week or longer) and incidence of graft failure, likely leading to BCVA decline [16].

An assumed predictive biomarker for graft detachment is the presence of stromal ripples, irregularities in the posterior corneal profile that assumed the shape of a ripple briefly after DMEK [22]. Coco et al. found that the presence of stromal ripples was significantly associated with the risk of graft detachments requiring rebubbling at any time, the risk of detachment of previously attached grafts, the risk of detachment worsening over time, and a higher CCT in mild or severe ripples compared with the absence of ripples [22]. In their study, OCT imaging was performed 2.9 ± 2.4 days after surgery. Consequently, when posterior stromal ripples are present, patients should be monitored more closely and managed on an individual basis as is the case with larger graft detachments [22]. Furthermore, stromal ripples were positively correlated with the CCT of patients after DMEK surgery [22]. However, whether they appeared first and lead to a problem with graft attachment or if they were a consequence of subclinical detachment is still not known [22]. While Coco et al. did not show if posterior stromal ripples were present before surgery, we looked for ripples in preoperative OCT images. In all three groups of our study, stromal ripples occurred prior to surgery. In some cases, complete resolution of severe stromal ripples occurred within 1 day after surgery; in others, an increase of severity over the course of 1 week was seen. One eye showed severe posterior stromal ripples both 1 day and 1 week after surgery. This very eye needed subsequent rebubbling. As our data reinforces, it remains uncertain if stromal ripples were the cause or consequence of a graft detachment. Further studies are needed to evaluate the cause of stromal ripples, and if preoperative or intraoperative factors influence their development [22].

As a limitation to our study, we chose to only include uncomplicated DMEK cases with limited concomitant diseases. That allowed us to reduce confounding factors yet limited the analysis of additional risk factors. Even though AS-OCT is the most common tool used for CCT measurement post DMEK, as it also allows the reliable visualization of graft detachments, other tools of CCT measurement such as ultrasound pachymetry or Scheimpflug camera (Pentacam) imaging have to be evaluated for their agreement with AS-OCT CCT values in attached and detached DMEK situations and for their suitability to predict different graft conditions, as we did in this study for AS-OCT.

If 1 week after uncomplicated DMEK CCT is < 600 µm and has decreased from before surgery, BCVA has improved, and there are no posterior stromal ripples, a graft detachment ≥ 1/3 and the need for rebubbling are very unlikely. In all other cases, meticulous slit-lamp and OCT inspection of the peripheral graft for detachments should be advised.
